# Elevating SOX2 Levels Deleteriously Affects the Growth of Medulloblastoma and Glioblastoma Cells

**DOI:** 10.1371/journal.pone.0044087

**Published:** 2012-08-28

**Authors:** Jesse L. Cox, Phillip J. Wilder, Michelle Desler, Angie Rizzino

**Affiliations:** Eppley Institute for Research in Cancer and Allied Diseases, University of Nebraska Medical Center, Omaha, Nebraska, United States of America; The University of Hong Kong, China

## Abstract

Medulloblastomas and glioblastomas are devastating tumors that respond poorly to treatment. These tumors have been shown to express SOX2 and overexpression of SOX2 has been correlated with poor prognosis. Although knockdown of SOX2 impairs the growth and tumorigenicity of brain tumor cells, it was unclear how elevating SOX2 levels would affect their fate. Interestingly, studies conducted with neural stem cells have shown that small increases or decreases in the level of this transcription factor significantly alter their fate. Here, we report that elevating SOX2 3-fold above endogenous levels in U87 and U118 glioblastoma, and DAOY medulloblastoma cells significantly impairs their ability to proliferate. We extended these findings and determined that elevating SOX2 in DAOY cells remodels their cell-cycle profile by increasing the proportion of cells in the G1-compartment, and induces the expression of genes associated with differentiation. Furthermore, we show that elevating SOX2 leads to a dramatic induction of CD133 expression in DAOY cells, yet inhibits the ability of both CD133^+^ and CD133^−^ cells to form neurospheres. Together, these findings argue that SOX2 levels must be carefully controlled in glioblastomas and medulloblastomas to maintain their fate. Equally important, our data suggests that increases in the expression of SOX2 during brain tumor progression are likely to be linked closely with changes in other critical genes that work in concert with SOX2 to enhance the tumorigenicity of brain tumors. Importantly, we demonstrate that this is also likely to be true for other cancers that express SOX2. Moreover, these studies demonstrate the advantage of using inducible promoters to study the effects of SOX2 elevation, as compared to gene expression systems that rely on constitutive expression.

## Introduction

Highly undifferentiated and aggressive brain tumors, including medulloblastoma and glioblastoma, are devastating diseases, which are difficult to treat. Glioblastoma multiforme, the most common and aggressive primary brain tumor, has a median survival time of 14 months despite considerable efforts to improve treatment [1]. Pediatric central nervous system tumors are the second most common malignancy in children (second only to leukemia), of which, medulloblastoma is the most common type. While the 5-year survival of medulloblastoma patients is nearly 80%, a treatment regime involving resection, radiotherapy and chemotherapeutics is associated with significant co-morbidities, including growth and endocrine abnormalities, as well as impairment of cognitive function [2], [3]. Moreover, the use of chemotherapeutics and radiation treatments predisposes young patients to future treatment-induced neoplasms and malignancy. Thus, new approaches are needed to improve the treatment of patients with medulloblastomas and glioblastomas.

Increased expression of the transcription factor SOX2 has been reported in a growing list of tumors, including breast, prostate, lung and in a number of highly aggressive central nervous system neoplasms, including both glioblastoma and medulloblastoma [4]–[8]. The expression and requirement of SOX2 in central nervous system tumors is not surprising, given that SOX2 is expressed in neural progenitor cells and their progeny [9]. In brain tumor initiating cells [10], [11], hypomethylation of the SOX2 promoter has been directly correlated with SOX2 expression, and the SOX2 promoter is hypomethylated in aggressive glioblastoma patient samples [12]. Additionally, it has recently been reported that the SOX2 gene is amplified in nearly 10% of glioblastomas and overexpressed in over 85% of these tumors [12]. Moreover, the knockdown of SOX2 has been shown to decrease the proliferation and tumorigenicity of glioblastomas [13].

SOX2 levels must be tightly controlled for proper development of the nervous system. In particular, perturbation of SOX2 levels in chick neural stem cells (NSC) has been shown to disrupt their fate. Aberrant elevation of SOX2 in chick NSC prevents their differentiation; whereas SOX2 knockdown induces the expression of gene markers of neural differentiation and causes the commitment of NSC to a differentiated fate [14], [15]. The careful regulation of SOX2 levels is also required to support the self-renewal of pluripotent, embryonic stem cells (ESC). In this regard, a 2-fold increase [16] or the knockdown of Sox2 [17] in mouse ESC disrupts their cell fate, causing ESC to lose their capacity for self-renewal and pluripotency.

Although NSC and ESC are adversely affected by increases in the levels of SOX2, other studies have reported that breast [18], prostate [19] and lung [20] cancer cells engineered to constitutively express elevated levels of SOX2 (∼3- to 4-fold) exhibit enhanced growth and tumorigenicity. Importantly, the cells in these reports were studied after selection for their incorporation of drug-resistant transgenes. Thus, these studies did not determine whether the majority of the cells in the tumor population, or only a minor fraction of cells, exhibit increase growth *in vitro* and *in vivo* in response to elevated levels of SOX2.

In view of the potent ability of SOX2 to dramatically alter the fate of NSC shortly following its elevation, we hypothesized that brain tumor cells must also tightly control the levels of SOX2 in order to support their growth. In this study, we demonstrate that the levels of SOX2 must be maintained below a certain threshold in glioblastoma and medulloblastoma. In this regard, we demonstrate that a 2- to 3-fold increase in SOX2 levels in the population of glioblastoma and medulloblastoma cells decreases their proliferation and alters their cellular morphology. We extended these findings by examining the effects of SOX2 elevation on the physiology of CD133^+^ and CD133^−^ subpopulations that are present in DAOY cells. We demonstrate that elevating SOX2 impairs the ability of both CD133^+^ and CD133^−^ DAOY cells to form neurospheres. We also determined that elevated SOX2 levels causes DAOY cells to exit the cell cycle and express gene markers associated with neural differentiation. Taken together, these studies demonstrate that glioblastoma and medulloblastoma cells must carefully control the expression of SOX2 to support their proliferation. Finally, we demonstrate similar effects in other types of tumors that express Sox2 endogenously. Two different breast tumor cell lines and a prostate tumor cell line each exhibit decreases in cell proliferation when SOX2 levels are elevated in the cell population from an inducible promoter. The implications of these findings are discussed, especially as they relate to increases in expression of SOX2 during cancer progression.

## Materials and Methods

### Cell Culture

DAOY medulloblastoma, U87 and U118 glioblastoma, MCF7 and MDA-231 breast cancer, and DU-145 prostate cancer cell lines were cultured in DMEM +10%, as described previously [21]. U87 cells and DU-145 cells were obtained from Webster Cavenee (Ludwig Institute for Cancer Research, La Jolla, CA) and Kaustabh Datta (University of Nebraska Medical Center, Omaha, NE), respectively, who obtained them from the American Type Culture Collection. All other cell lines were obtained directly from the American Type Culture Collection.

### Plasmid Production

Lentiviral packaging vector psPAX2 (Plasmid 12260), envelope vector pMD2.G, (Plasmid 12259), and transfer vectors FUW-M2rtTA (Plasmid 20342) and doxycycline (Dox)-inducible FUW-tetO-SOX2 (Plasmid 20724) were obtained from Addgene (Cambridge, MA). G418-selectable pLVX-Tet-On® Advanced vector was obtained from Clontech (632162, Mountain View, CA). Construction of pLVX-tetO-(fs)SOX2 is described in Methods S1.

### Lentivirus Production and Cell Transduction

Production of lentiviruses and cell transduction has been described previously [22]. After culture in medium containing lentiviruses for 24 hours, the cells were refed with fresh medium. For cells infected with shRNA lentiviruses, cells were selected in medium containing 5 µg/mL puromycin (P8833, Sigma-Aldrich, St. Louis, MO) for 24 hours.

### Photomicrographs, MTT Cell Growth Assay and Determination of Cell Number

DAOY, U87, U118, MCF7 and MDA-231 cells were subcultured into 12-well plates, transduced with appropriate lentiviruses, and refed with fresh medium containing the indicated concentration of Dox. At the time points indicated, photomicrographs were taken with a Canon Rebel XTi camera, and MTT assays were conducted. The protocol for the MTT assay, which uses mitochondrial dehydrogenase activity as a measure of cell number, was described previously [23], [24]. Where indicated cell number was also determined with the aid of a Beckman Coulter Counter.

### Nuclear Protein Isolation and Western Blot Analysis

Nuclear proteins were isolated from DAOY, U87, U118, MCF7 and MDA-231 cells using the NE-PER™ kit (Pierce, Rockford, IL), as described previously [25]. Isolated proteins were quantified using a Micro BCA Kit (Pierce), according to the manufacturer’s protocol. Equal amounts of nuclear protein were separated by SDS-PAGE and blotted as described previously [25]. The primary antibodies used were α-SOX2 (ab15830, Abcam, Cambridge, MA) and α-FLAG (F1804, Sigma-Aldrich); the secondary antibodies were α-Rabbit IgG-AP conjugate (A3687, Sigma-Aldrich) and α-Mouse IgG-AP conjugate (A4312, Sigma-Aldrich). Relative protein abundance was quantified using ImageQuant 5.2, and small differences in loading were corrected by comparison to the loading control, which was HDAC1 where indicated or a ubiquitously expressed non-specific protein band.

### Generation of i-SOX2-DAOY and i-SOX2-U87 Cells

DAOY and U87 cells were transduced for 24 hours with both pLVX-Tet-On® Advanced and pLVX-tetO-(fs)SOX2 lentiviral vectors. Transduced cells were selected by maintaining cells in culture medium supplemented with 5 µg/mL puromycin for 48 hours. Following puromycin selection, cells were maintained in culture medium supplemented with 300 ng/mL G418 sulfate (631307, Clontech) for 9 days.

### Cell-Cycle Analysis

i-SOX2-DAOY cells were infected as described above, cultured in fresh medium containing Dox for 72 hours, and isolated for cell-cycle analysis. The Telford method was used [26], and FACS analysis was conducted by the UNMC Cell Analysis core facility.

### RNA Isolation, RT-qPCR Analysis and Microarray Analysis

RNA isolation and cDNA synthesis were performed as described previously [16]. Primer sets for RT-qPCR are listed in Table S1. GeneChip Human Gene 1.0 ST arrays were used for microarray analysis (901085, Affymetrix, Santa Clara, CA). A single Affymetrix chip was used for cells cultured without Dox, and a single chip was used for cells cultured with Dox. Array data has been deposited in the Gene Expression Omnibus repository (Accession No. GSE36947, available at http://www.ncbi.nlm.nih.gov/geo/query/acc.cgi?acc=GSE36947).

### CD133/SSEA1 Detection and Flow Cytometry

Cells were collected, washed twice in stain buffer (0.2% (w/v) bovine serum albumin in PBS), and resuspended at 10^6^ cells/100 µL. The cell suspension was supplemented with antibody and incubated for 20 minutes on ice in the dark. Next, the cells were washed twice and resuspended in 1 mL stain buffer. Cells were then FACS sorted/analyzed. The following antibodies were used: α-CD133-APC (10 µL/sample, 130-090-854, Miltenyi Biotech, Auburn, CA) and α-SSEA1-FITC (20 µL/sample, 560127, BD Pharmingen, San Diego, CA). Annexin V was detected with a TACS® Annexin V-FITC Kit (4830-01-K, Trivigen, Inc., Gaithersburg, MD).

### Neurosphere Growth

For neurosphere culture, cells were cultured in bacterial petri dishes in DME/F12 supplemented with 2% B27 (12587010, Invitrogen, Carlsbad, CA), 1% Glutamax (35050061, Invitrogen), 20 ng/mL EGF (E9644, Sigma-Aldrich), 20 ng/mL FGF2 (F0291, Sigma-Aldrich), 20 ng/mL LIF (ESG1107, Millipore, Billerica, MA) and the indicated concentrations of Dox. The cells were observed daily.

## Results

### Ectopic SOX2 Expression Impairs Glioblastoma Cell Growth

A number of cancers, including glioblastomas, medulloblastomas and breast cancers, express SOX2, which has been correlated with poorer prognosis and clinical outcome [4]–[8]. While Gangemi and colleagues demonstrated that a reduction of SOX2 in glioblastoma tumor cells leads to a significant reduction in cell proliferation [13], it was unclear how glioblastoma tumor cells would respond to rapidly elevated levels of SOX2. To address this question, we initially examined the impact of elevating SOX2 on the physiology of glioblastoma cells.

**Figure 1:**
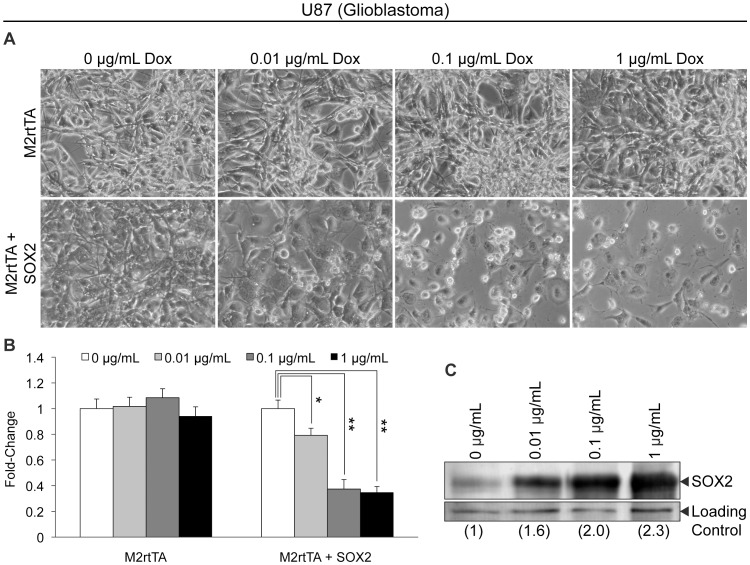
Ectopic elevation of SOX2 in U87 glioblastoma cells. (A) Photomicrographs of U87 cells infected with FUW-M2rtTA or FUW-M2rtTA and FUW-tetO-SOX2 lentiviruses. Photomicrographs were taken 48 hours after the addition of Dox to the culture medium, which induces ectopic SOX2 expression. (B) MTT assay of U87 glioblastoma cells, infected with FUW-M2rtTA or FUW-M2rtTA and FUW-tetO-SOX2 lentiviruses, cultured in various concentrations of Dox for 48 hours. Triplicates of each condition tested were averaged, and the error bars represent standard deviations. MTT values of cells cultured without Dox were set to one. This experiment was repeated two additional times, and similar results were obtained in each case. ‘*’ and ‘**’ indicate statistical significance (p<0.01 and p<0.001, respectively, student’s t-test). (C) Western blot analysis of SOX2 protein levels in nuclear extracts from U87 cells infected with FUW-M2rtTA and FUW-tetO-SOX2 lentiviruses, cultured without or with Dox for 24 hours to induce SOX2 expression.

U87 glioblastoma cells were engineered to enable the inducible expression of SOX2 controlled by a tet-responsive element, which could be activated by adding doxycycline (Dox) to the medium. For this purpose, U87 cells were transduced with the FUW-M2rtTA lentiviral vector alone (to introduce a constitutively expressed tet-responsive element) or in combination with the lentiviral vector FUW-tetO-SOX2 (to introduce an ectopic SOX2 transgene under the control of a tet-responsive promoter). Following transduction, cells were refed with culture medium supplemented with increasing concentrations of Dox, and cells were monitored daily. Following 48 hours of SOX2 induction, U87 cell density was dramatically reduced as compared to controls without Dox (Figure 1A). Moreover, elevation of SOX2 led to substantial changes in cell morphology and loss of spindle shaped cells commonly observed in U87 cultures. MTT assays demonstrated that elevated SOX2 significantly reduced cell number at each Dox concentration tested (65% reduction at the highest concentration of Dox tested) (Figure 1B). Importantly, this reduction was not observed in U87 cells transduced with the FUW-M2rtTA vector alone. Remarkably, these dramatic changes in U87 cell physiology occurred following modest increases in SOX2 (2.3-fold) (Figure 1C).

To determine whether the dramatic phenotypic changes observed in U87 cells upon ectopic SOX2 expression could be extended to other glioblastoma lines, we performed similar analyses in U118 glioblastoma cells. As in the case of U87 cells, lentiviral vectors were used to deliver FUW-M2rtTA alone or in combination with FUW-tetO-SOX2. Ectopic expression of SOX2 for 48 hours resulted in a dramatic reduction in proliferation and survival of U118 cells, as determined by microscopy observations (Figure S1A). MTT assays demonstrated that elevation of SOX2 led to a significant, dose-dependent reduction in U118 cell number at each Dox concentration tested (Figure S1B). Quantitation of SOX2 levels in U118 glioblastoma cells demonstrated that SOX2 levels rose 2.1-fold one day after addition of Dox to the culture medium (Figure S1C). Together, these findings argue that modest increases (2- to 3-fold) in SOX2 levels substantially disrupt the *in vitro* growth of glioblastoma cells and cause dramatic changes in their cell morphology. Thus, SOX2 levels must be carefully maintained within these cells.

### Elevation of SOX2 in Medulloblastoma Cells Impairs their Ability to Proliferate

**Figure 2:**
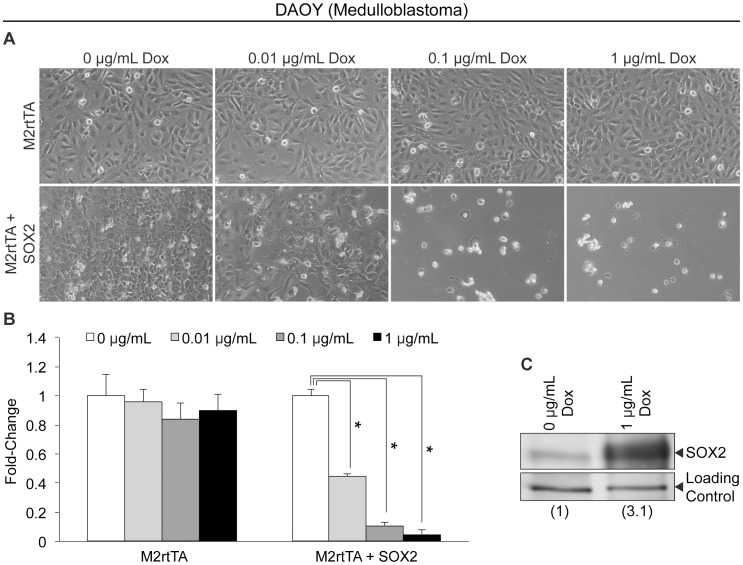
Ectopic elevation of SOX2 in DAOY medulloblastoma cells. (A) Photomicrographs of DAOY cells infected with FUW-M2rtTA or FUW-M2rtTA and FUW-tetO-SOX2 lentiviruses, cultured in the presence of Dox, at the indicated concentration, for 48 hours. (B) MTT assay of DAOY medulloblastoma cells infected with FUW-M2rtTA or FUW-M2rtTA and FUW-tetO-SOX2 lentiviruses, cultured in various concentrations of Dox for 48 hours. Triplicates of each condition tested were averaged, and the error bars represent standard deviations. MTT values of cells cultured without Dox were set to one. This experiment was repeated two additional times, and similar results were obtained in each case. ‘*’ indicates statistical significance (p<0.001, student’s t-test). (C) Western blot analysis of SOX2 protein levels in nuclear extracts from DAOY cells infected with FUW-M2rtTA and FUW-tetO-SOX2 lentiviruses, cultured without or with Dox for 24 hours to induce SOX2 expression.

The effects of elevating SOX2 in glioblastoma led us to examine whether elevating SOX2 also influences the growth of medulloblastoma tumor cells. For this purpose, DAOY medulloblastoma cells were transduced with the FUW-M2rtTA lentiviral vector alone, or in combination with the FUW-tetO-SOX2 vector described above. Similar to the glioblastoma cells, transduced DAOY cells were cultured in the presence of increasing concentrations of Dox to induce SOX2 expression. Following SOX2 elevation, DAOY cell density was dramatically reduced, as determined by microscopy observations (Figure 2A). Moreover, MTT assays were used to quantify the effects of SOX2, and significant, dose-dependent declines in cell viability were observed (90% reduction at the highest concentration of Dox tested) (Figure 2B). Western blot analysis revealed a ∼3-fold increase in total SOX2 levels at 1 µg/mL Dox compared to endogenous levels in the absence of Dox (Figure 2C). The differences observed in the sensitivity of these brain tumor cell lines to exogenous SOX2 (introduced by lentiviral transduction) is likely to be due, at least in part, to differences in the transduction efficiencies of the various cell lines. In this regard, DAOY cells are infected at a higher frequency (∼90%) than U118 cells (∼63%), as determined by transduction with GFP-expressing lentiviruses and FACS analysis (data not shown). Together, our findings argue that the levels of SOX2 must be carefully controlled to support the growth of U87 and U118 glioblastoma cells, as well as DAOY medulloblastoma cells.

### Elevating SOX2 Levels Impedes the Growth of both Prostate and Breast Tumor Cells

**Figure 3:**
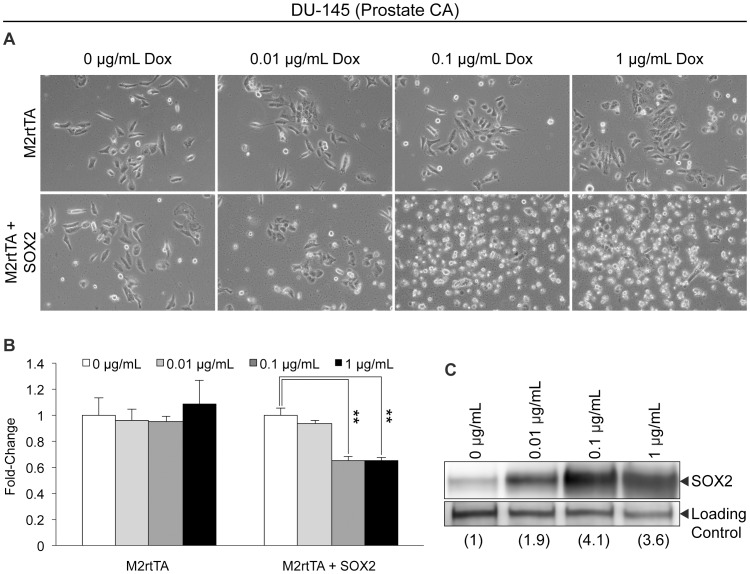
Ectopic elevation of SOX2 in DU-145 prostate cancer cells. (A) Photomicrographs of DU-145 prostate cancer cells infected with FUW-M2rtTA or FUW-M2rtTA and FUW-tetO-SOX2 lentiviruses, cultured in the presence of Dox, at the indicated concentration, for 48 hours. (B) MTT assay of DU-145 prostate cancer cells infected with FUW-M2rtTA or FUW-M2rtTA and FUW-tetO-SOX2 lentiviruses, cultured in various concentrations of Dox for 48 hours. Triplicates of each condition tested were averaged, and the error bars represent standard deviations. MTT values of cells cultured without Dox were set to one. This experiment was repeated two additional times, and similar results were obtained in each case. ‘**’ indicates statistical significance (p<0.001, student’s t-test). (C) Western blot analysis of SOX2 protein levels in nuclear extracts from DU-145 cells infected with FUW-M2rtTA and FUW-tetO-SOX2 lentiviruses, cultured without or with Dox for 24 hours to induce SOX2 expression.

Our findings with glioblastoma and medulloblastoma cells differ from those in other studies where it was reported that elevating SOX2 enhances the growth and tumorigenicity of DU145 prostate tumor cells [19], MCF7 breast tumor cells [18] and A549 lung tumor cells [20]. To determine whether the impairment of cell growth following SOX2 elevation is exclusive to brain tumor cells, we used the same experimental approach described above for brain tumor cells to examine the response of prostate cancer cells to elevation of SOX2. Prostate cancer cells (DU-145) were transduced with lentiviral vectors FUW-M2rtTA and FUW-tetO-SOX2 to introduce a constitutively expressed reverse-tet transactivator and a SOX2 transgene under the control of a tet-responsive element, respectively. Following transduction, cells were cultured for 48 hours in medium supplemented with Dox to induce exogenous SOX2 expression (Figure 3C). Interestingly, when SOX2 levels were rapidly induced in DU-145 cells, a marked, dose-dependent reduction in the number of viable cells was observed by both light microscopy (Figure 3A) and MTT assay (Figure 3B).

**Figure 4:**
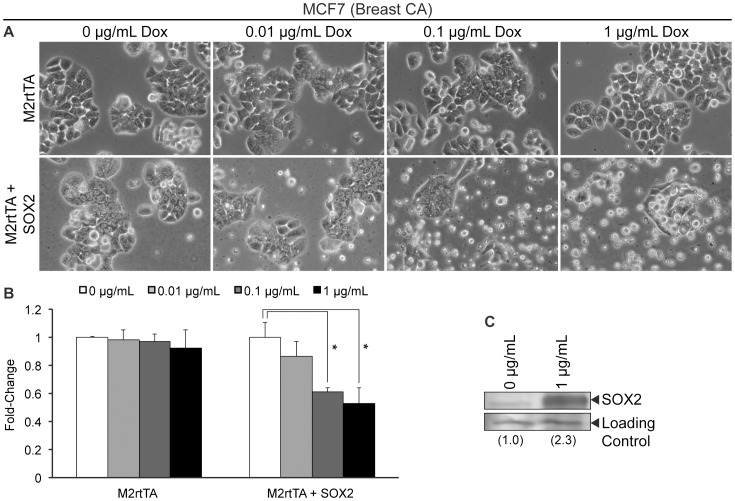
Ectopic elevation of SOX2 in MCF7 breast cancer cells. (A) Photomicrographs of MCF7 breast cancer cells infected with FUW-M2rtTA or FUW-M2rtTA and FUW-tetO-SOX2 lentiviruses, cultured in the presence of Dox, at the indicated concentration, for 48 hours. (B) MTT assay of MCF7 breast cancer cells infected with FUW-M2rtTA or FUW-M2rtTA and FUW-tetO-SOX2 lentiviruses, cultured in various concentrations of Dox for 48 hours. Triplicates of each condition tested were averaged, and the error bars represent standard deviations. MTT values of cells cultured without Dox were set to one. This experiment was repeated two additional times, and similar results were obtained in each case. ‘*’ indicates statistical significance (p<0.01, student’s t-test). (C) Western blot analysis of SOX2 protein levels in nuclear extracts from MCF7 cells infected with FUW-M2rtTA and FUW-tetO-SOX2 lentiviruses, cultured without or with Dox for 24 hours to induce SOX2 expression.

We also examined whether a rapid elevation of SOX2 could perturb the growth of breast cancer cells. For this purpose, MCF7 and MDA-231 cells were transduced with lentiviral vectors FUW-M2rtTA and FUW-tetO-SOX2. Following transduction, cells were cultured for 48 hours in medium supplemented with Dox to induce exogenous SOX2 expression. The increase in SOX2 levels induced in MCF7 (Figure 4) and MDA-231 (Figure S2) cells (∼2- to 3-fold) were similar to the levels of SOX2 in MCF7 cells engineered to stably overexpress SOX2 [18], as well as the level of SOX2 induction observed in our brain tumor studies presented above. In contrast to the previous report [18], we observed a notable reduction in cell number by light microscopy, 48 hours after SOX2 induction, in both MCF7 (Figure 4) and MDA-231 (Figure S2) breast cancer cells. Moreover, we observed a dose-dependent reduction in the cell population after the induction of SOX2 expression, demonstrated by MTT assay, in both MCF7 (Figure 4) and MDA-231 (Figure S2). Thus, the impairment of tumor cell growth following elevation of SOX2 is not unique to brain tumor cells. Moreover, the different conclusions reached here and in the former report [18] likely arise from differences in experimental design, as discussed more fully later in this report.

### Elevating SOX2 Levels in DAOY Cells Modulates the Expression of the Cell Surface Marker CD133

**Figure 5:**
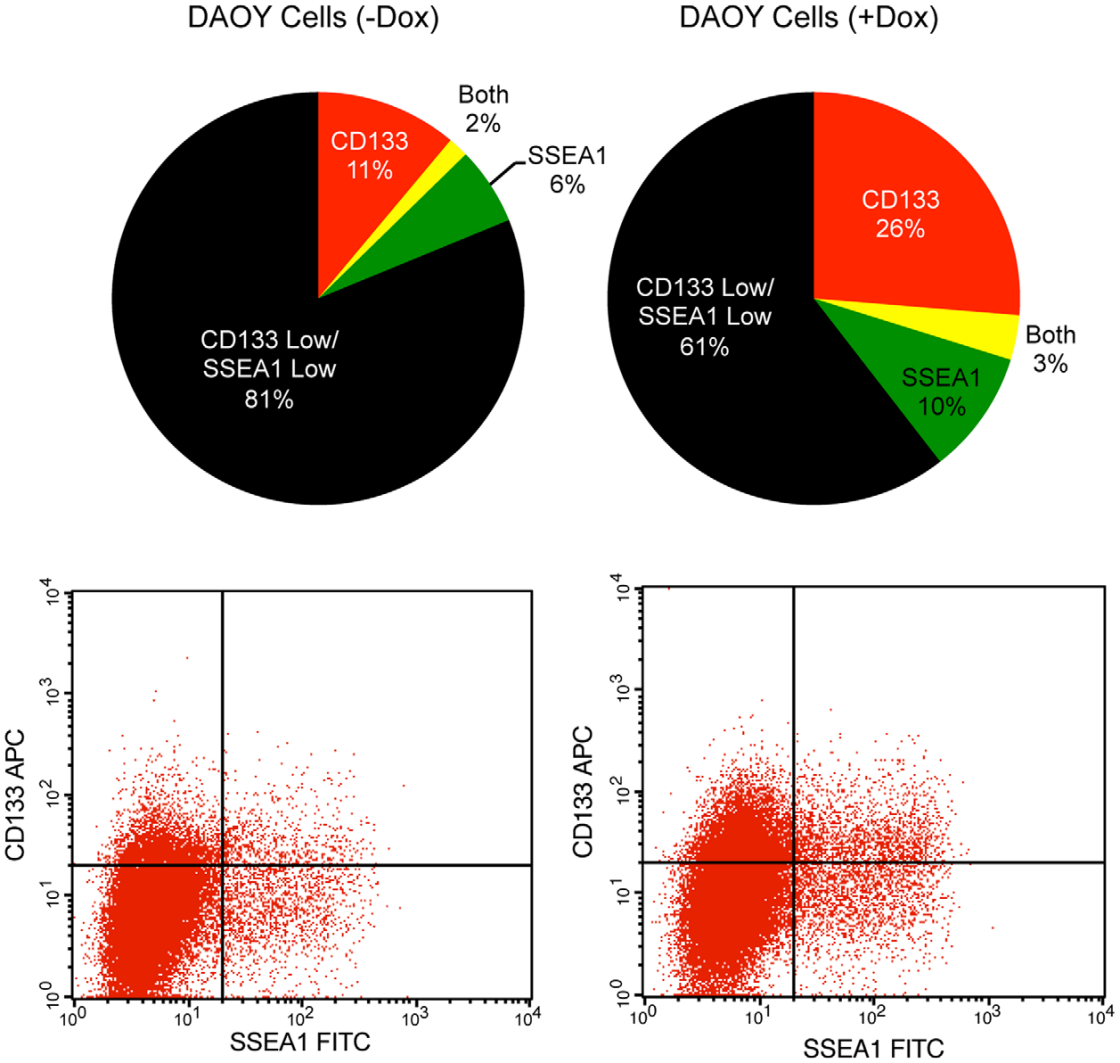
Examination of cell surface markers SSEA1 and CD133 following exogenous expression of SOX2 in DAOY medulloblastoma cells. DAOY cells were infected with FUW-M2rtTA and FUW-tetO-SOX2 lentiviruses and cultured without or with Dox (1 µg/mL). Twenty-four hours after the addition of Dox, cells were collected and examined for SSEA1 and CD133 surface expression by FACS analysis, as described in the Materials and Methods.

Cell lines, like *in vivo* tumors, are composed of a heterogeneous population of cells. To determine whether SOX2 may affect subpopulations within the DAOY cell line differently, we initially examined the surface expression of CD133 and SSEA1 with and without the elevation of SOX2. CD133 has been implicated in a number of cancers as a potential marker for identifying tumor-initiating cells, which are capable of reforming the original histopathology of the tumor [10], [27], [28]. SSEA1 has also been shown to identify a tumor-initiating cell population in brain tumors [29]. To examine the distribution of CD133^+^ and/or SSEA1^+^ cells following the elevation of SOX2, DAOY cells were transduced with both the FUW-M2rtTA and FUW-tetO-SOX2 lentiviral vectors. After transduction, the cells were exposed to 1 µg/mL of Dox for 24 hours, and harvested for two-color flow analysis as described in Materials and Methods. Exogenous expression of SOX2 caused an expansion of CD133^+^ and SSEA1^+^ populations, ∼2.5-fold and 50%, respectively (Figure 5). However, it was unclear whether this increase in the proportion of CD133^+^ and SSEA1^+^ cells was due to deleterious effects of SOX2 on CD133^−^/SSEA1^−^ cells, or to induction of CD133/SSEA1 expression.

Because the proportion of CD133^+^/SSEA1^+^ cells increased following elevation of SOX2, we examined the ability of DAOY cells to form neurospheres when SOX2 is elevated. Anchorage-independent growth is often used to assess the tumor-initiating potential of a cell population *in vitro* [27]. As expected, DAOY cells formed neurospheres in the absence of Dox (Figure S3). In contrast, elevating SOX2 dramatically reduced the capacity of DAOY cells to grow in suspension. Importantly, this observation, along with additional studies presented below, argues that SOX2 impairs the ability of both CD133^+^ and CD133^−^ DAOY cells to form neurospheres.

### Generation of i-SOX2-DAOY Medulloblastoma Cells

**Figure 6:**
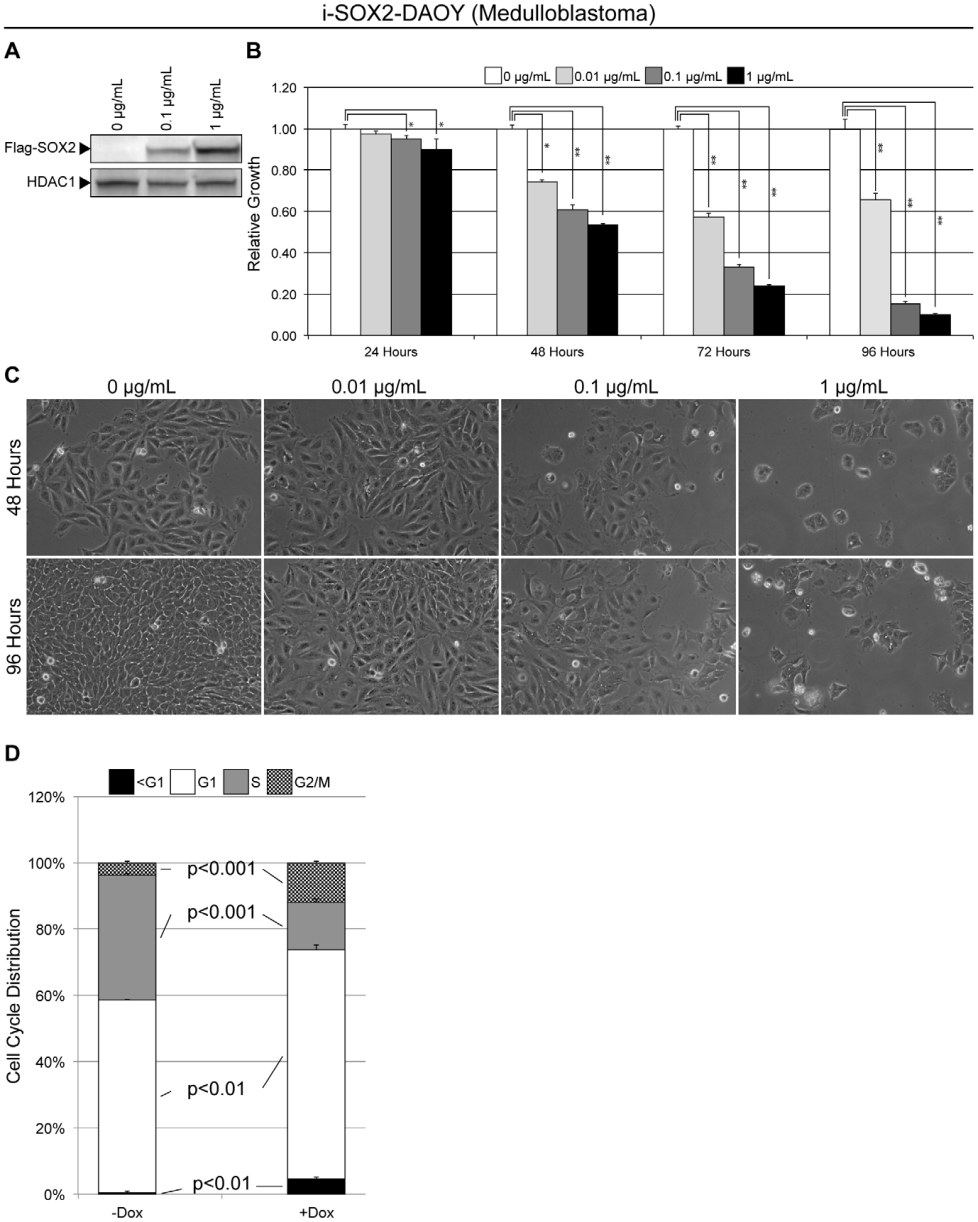
Characterization of i-SOX2-DAOY medulloblastoma cells. (A) i-SOX2-DAOY cells were exposed to Dox for 24 hours and nuclear extracts were isolated. A Flag-antibody was used to probe a western blot for the expression of flag-epitope tagged SOX2. (B) MTT assay of i-SOX2-DAOY cells after various duration exposure to Dox. Triplicates of each condition tested were averaged, and the error bars represent standard deviations. ‘*’ and ‘**’ indicates statistical significance (p<0.05 and p<0.005, respectively, student’s t-test). (C) Photomicrographs of i-SOX2-DAOY cells following 48 and 96 hours exposure ± Dox (1 µg/mL). (D) Cell cycle analysis of i-SOX2-DAOY cultured in the absence or presence of Dox (1 µg/mL) for 48 hours. Data for cells without Dox was collected as independent duplicates; whereas, samples with Dox were collected in triplicate. Error bars represent standard deviation. The student’s t-test was used to determine p-values.

Although lentiviral transduction of DAOY medulloblastoma was highly efficient (data not shown), the lentiviral vectors used above to characterize the response of brain tumor cells are not well suited to detailed examination of larger numbers of cells, especially when one wishes to examine many different cellular parameters. Consequently, we engineered DAOY cells for the inducible expression of epitope tagged SOX2 using lentiviral vectors containing selection markers, as described in the Materials and Methods. This generated a stably transduced cell population, referred to as i-SOX2-DAOY cells, which express Flag-tagged SOX2 in the presence of Dox (Figure 6A). Importantly, these cells responded to Dox-driven SOX2 induction similarly to our earlier cell populations. Microscopic observations demonstrated that elevation of SOX2 reduced the growth of these cells in a dose-dependent manner, which became clear while following their growth over 4 days. This was confirmed using an MTT assay (Figure 6B).

We used i-SOX2-DAOY cells to examine the cell cycle distribution following the induction of SOX2. Elevation of SOX2 in i-SOX2-DAOY cells resulted in significant enrichment of the G2/M (4% to 12%) and G1 compartments (58% to 69%), and a significant decrease in cells in S-phase (38% to 14%) (Figure 6D). Additionally, we observed an increase in the sub-G1 compartment; however, western blot analysis and FACS analysis did not reveal significantly increased levels of cleaved Parp or Annexin V staining, respectively, following 48 hours of ectopic SOX2 expression (data not shown). We also examined β-galactosidase activity, which is reported to increase when cells become senescent [30]; however, we did not detect increased β-galactosidase staining following ectopic SOX2 elevation (data not shown). Thus, i-SOX2-DAOY cells exited the cell cycle (Figure 6D), and likely became quiescent during the time period examined.

### Exogenous SOX2 Expression Slows the Long-term Growth of Brain Tumor Cells

From previous reports demonstrating that elevation of SOX2 can enable certain tumor cells to grow more rapidly, we postulated that, given enough time in the presence of elevated SOX2, a subpopulation of tumor cells could emerge which expressed exogenous SOX2 and proliferates rapidly. To address this possibility, we used i-SOX2-DAOY cells to examine the long-term effects of SOX2 elevation on the growth of DAOY medulloblastoma cells. For this purpose, i-SOX2-DAOY cells, cultured in the absence and presence of Dox, were passaged at clonal density every week for four weeks. Although i-SOX2-DAOY cells were able to proliferate slowly and form small colonies, the growth of i-SOX2-DOAY cells remained low when SOX2 was elevated, even after 1 month in culture (Figure S4).

To determine whether exogenous SOX2 expression could impair the long-term growth of other brain tumors types, we stably engineered U87 glioblastoma cells for Dox-inducible expression of SOX2 (i-SOX2-U87, Figure S5) using the same lentiviral vectors used to engineer i-SOX2-DAOY cells. We first characterized the short-term effects of SOX2 elevation on i-SOX2-U87 cells. Similar to our initial observations in U87 cells (Figure 1), elevation of SOX2 in i-SOX2-U87 cells (Figure S5A) led to a dramatic morphological change (Figures S5B & C) and a reduction in cell number (Figure S5D). Moreover, exogenous SOX2 elevation caused a significant change in the cell cycle distribution of i-SOX2-U87 cells. These cells exhibited an increase in the G2/M cell cycle compartment (10% to 17%), a reduction in the proportion of cells in the G1 compartment (75% to 64%), and a dramatic increase in cells in the sub-G1 compartment (0.14% to 11%), indicative of cells undergoing apoptosis (Figure S5E). To confirm that i-SOX2-U87 cells were undergoing apoptosis following elevation of SOX2 levels, annexin V staining and FACS analysis was conducted. Similar to our cell cycle analysis, annexin V positive i-SOX2-U87 cells increased in the presence of elevated SOX2 levels from 2.2% to 9.2% (Figure S5F).

We then examined whether exogenous SOX2 expression would impair the growth of i-SOX2-U87 cells for a longer period in culture (e.g. 4 weeks), similar to our observations in i-SOX2-DAOY cells (Figure S4). i-SOX2-U87 cells were passaged weekly at clonal density for 4 weeks in total. Similar to our findings in i-SOX2-DAOY cells, elevation of SOX2 in i-SOX2-U87 cells for 1 month impaired their growth dramatically at clonal densities (Figure S6). Thus, both medulloblastoma and glioblastoma cells cannot readily overcome the effects of elevated SOX2 levels, even after 4 weeks in culture. Taken together, although both DAOY and U87 exhibited some proliferative capacity in the presence of exogenous SOX2, i-SOX2-DAOY and i-SOX2-U87 cells were unable to proliferate at the same rate as their uninduced counterparts, even after 4 weeks of culture with elevated SOX2 levels.

### Exogenous SOX2 Induces CD133 Expression and Gene Markers of Neural Differentiation

**Figure 7:**
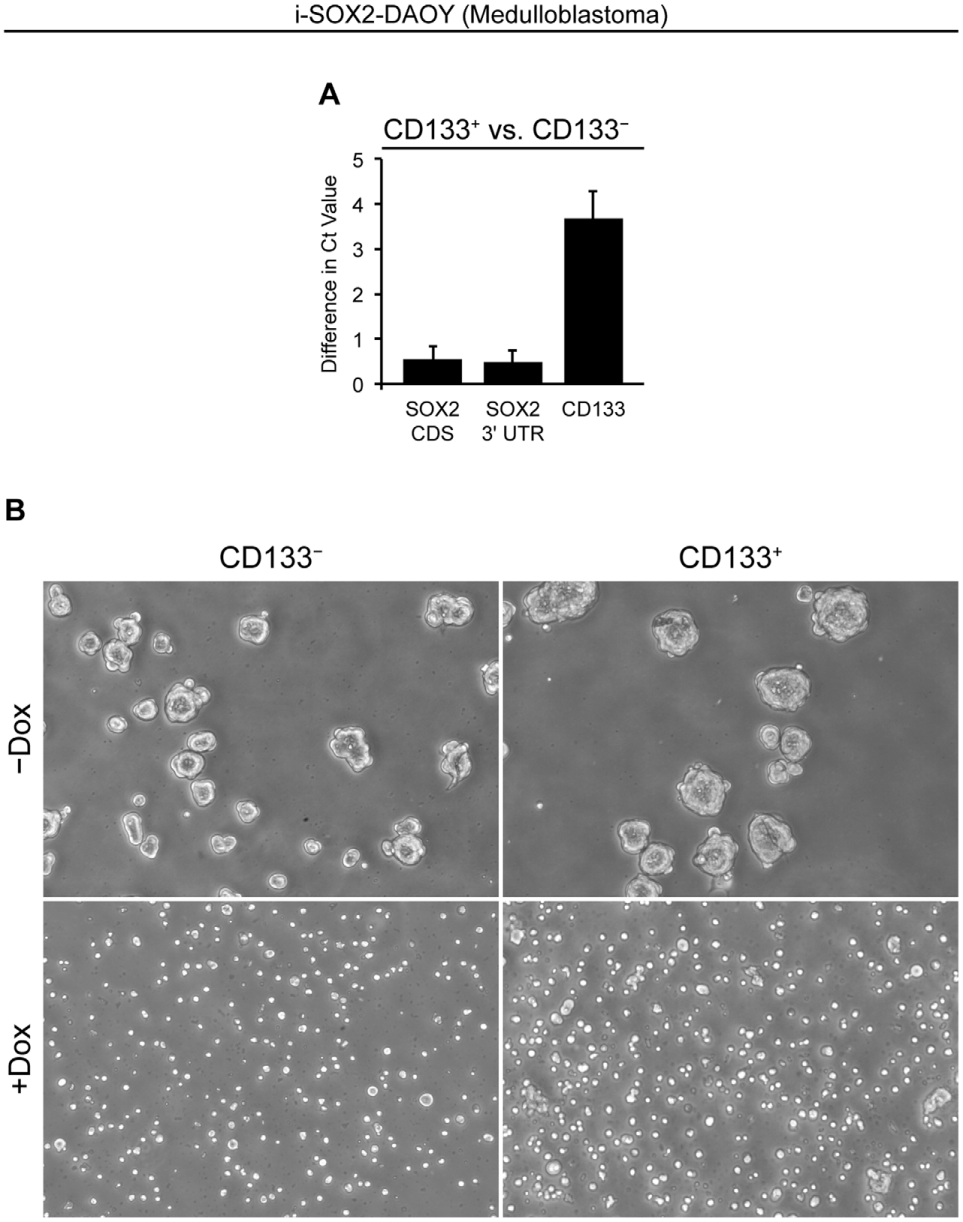
Neurosphere culture of CD133^+^ and CD133 ^−^ **i-SOX2-DAOY Cells in the absence and presence of Dox.** i-SOX2-DAOY cells were FACS sorted into CD133^−^ and CD133^+^ populations. SSEA1^+^ cells were excluded from both populations. (A) RNA was isolated from sorted cells, cDNA was prepared and RT-qPCR was conducted. Values represented are differences in Ct value (normalized by correcting for differences in Ct values of GAPDH), where a positive value indicates increased expression of a given transcript within the CD133^+^ population. (B) Freshly sorted i-SOX2-DAOY cells were placed into suspension culture in the absence or presence of Dox (1 µg/mL), as described in the Materials and Methods. Photomicrographs were taken after 5 days in culture.

To more fully characterize the impact of SOX2 on the expression of CD133 in medulloblastoma cells, i-SOX2-DAOY cells (not exposed to Dox) were sorted into CD133-high (CD133^+^) and CD133-low (CD133^−^) populations by FACS. RNA was then immediately isolated from both populations. RT-qPCR analysis verified that the CD133^−^ cell population was virtually devoid of CD133 transcript, especially when compared to the CD133^+^ population (Figure 7A). Interestingly, levels of SOX2 are slightly higher in the CD133^+^ population compared to their CD133^−^ counterparts in DAOY cells, similar to a previous report regarding glioblastoma cells [31]. At the same time, we also cultured the two cell populations on tissue culture plastic (monolayer culture) in medium supplemented with increasing concentrations of Dox. After exposure for 48 hours, both populations exhibited a reduction in cell number when Dox was added (data not shown). In addition, we examined the two cell populations for their ability to form neurospheres in suspension culture. Remarkably, there was no difference in the ability of the i-SOX2-DAOY CD133^−^ and CD133^+^ populations to form neurospheres. Moreover, both populations exhibited dramatic reductions in their ability to form neurospheres when Dox was added (Figure 7B), arguing that CD133^−^ DAOY cells as well as the putative tumor-initiating population of CD133^+^ DAOY cells must carefully regulate SOX2 levels to support their self-renewal.

**Figure 8:**
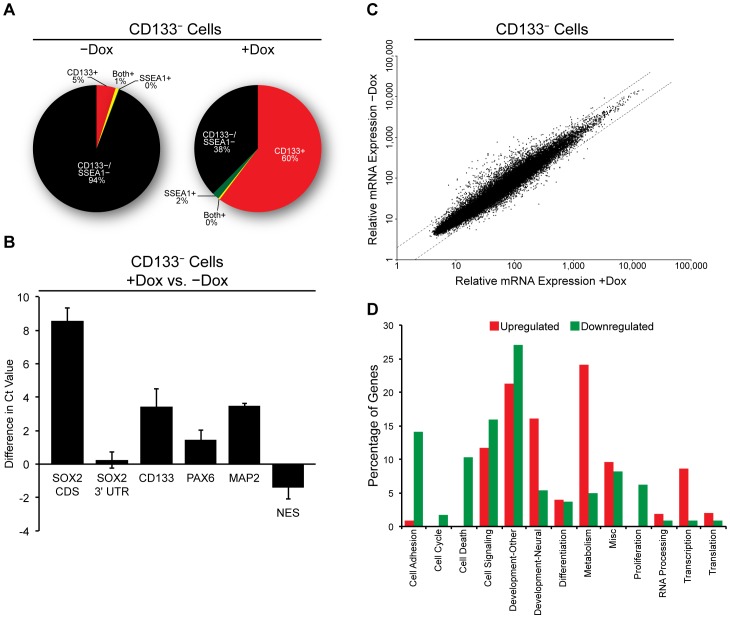
Effects of SOX2 overexpression on CD133 ^−^ **DAOY cells.** i-SOX2-DAOY cells were FACS sorted into CD133^−^ and CD133^+^ populations. SSEA1^+^ cells were excluded from both populations. (A) CD133^−^ cells were cultured in medium without or with Dox (0.5 µg/mL) for 24 hours. Cells were then collected for FACS analysis of surface expression of CD133 and SSEA1. (B) RNA was isolated from CD133^−^ cells cultured without and with Dox (0.5 µg/mL) for 24 hours. cDNA was prepared and RT-qPCR was conducted. Values represented are differences in Ct value (normalized as described in Figure 7), where a positive value indicates increased expression of a given transcript within the Dox supplemented population. (C) A scatter plot of transcript expression, as determined by Affymetrix microarray analysis, comparing i-SOX2-DAOY cells cultured in the absence or presence of Dox (0.5 µg/mL) for 24 hours. Transcript expression from CD133^−^ DAOY cells cultured without Dox is represented on the y-axis; whereas, transcript expression from cells cultured with Dox is represented on the x-axis. A single replicate of each condition was analyzed. RT-qPCR analysis (subfigure B) was used to validate the array data. (D) Gene ontology analysis comparing genes that were upregulated or genes that were downregulated 2-fold, in CD133^−^ DAOY cells cultured in the absence or presence of Dox. The y-axis represents the percentage of up- or downregulated transcripts that reside within various ontology categories.

Our observations suggested that the overall increase in CD133 expression following SOX2 elevation (Figure 5) is due to the upregulation of CD133 surface expression, and not due to the ability of SOX2 to preferentially impair the growth of CD133^−^ cells. To test this possibility more directly, CD133^−^ i-SOX2-DAOY cells were isolated and placed in culture in the absence and the presence of Dox. Twenty-four hours after sorting, samples of these cells were subjected to FACS analysis to examine CD133 expression (Figure 8A). As noted above, CD133^−^ i-SOX2-DAOY cells expressed very low levels of CD133 transcripts. Surprisingly, within only 24 hours, the population grown without Dox had begun to re-express cell surface CD133. Even more remarkable, treatment with Dox caused a large increase in the percentage of CD133^+^ cells, rising from ∼5% to ∼60%.

To verify that SOX2 was capable of increasing CD133 transcript levels in CD133^−^ i-SOX2-DAOY cells and to characterize the transcript profile of these cells further, we examined how SOX2 altered the gene expression profile of CD133-low i-SOX2-DAOY cells. For this purpose, FACS sorted CD133^−^ i-SOX2-DAOY cells were cultured with or without Dox for 24 hours. At that point, RNA was isolated from the two populations. RT-qPCR analysis (Figure 8B) indicated that treatment with Dox caused a large increase in the expression of the SOX2 coding sequence (exogenous plus endogenous) in CD133^−^ i-SOX2-DAOY cells, but there was little or no difference in the level of endogenous transcripts containing the SOX2 3′UTR. Additionally, transcript levels of CD133 were elevated significantly and exhibited an increase of more than three cycle-threshold values (Ct Values). Thus, exogenous expression of SOX2 induces the expression of the cell surface marker CD133 at both the RNA level (Figure 8B) and at the cell surface expression level (Figure 8A).

To gain deeper insight into the changes in gene expression induced by elevation of SOX2, RNA isolated from i-SOX2-DAOY cells, cultured in the absence or presence of Dox for 24 hours, was subjected to Affymetrix microarray analysis (Table S2, Accession No. GSE36947, available at http://www.ncbi.nlm.nih.gov/geo/query/acc.cgi?acc=GSE36947). Scatter plot analysis of the microarray data demonstrated significant changes in the gene expression profile of DAOY cells following the induction of SOX2 (Figure 8C). Of 8,518 transcripts represented on the microarray, 1.67% (142) were upregulated and 5.61% (478) were downregulated ≥2-fold. Gene ontology classification and analysis was conducted using the Database for Annotation, Visualization and Integrated Discovery (DAVID) to characterize the transcripts and biological processes changed ≥2-fold by elevation of SOX2 [32], [33]. Genes associated with cell-adhesion, cell death and cell proliferation, were largely downregulated; whereas, genes associated with neural development, metabolism and transcription were upregulated (Figure 8D).

To confirm our microarray results, transcript levels of genes used as markers for neural development were examined more closely using RT-qPCR. As expected, PAX6 and MAP2, markers of neuronal differentiation [34], [35], rose (Figure 8B); whereas, transcript levels of Nestin (NES), a neural progenitor cell marker and a marker of general central nervous system development, decreased upon exogenous SOX2 expression (Figure 8B). The changes observed in markers associated with neural development align with our observations above demonstrating that iSox-DAOY accumulate in the G1-cell cycle compartment and become quiescent (Figure 6D). Moreover, our RT-qPCR analysis closely aligned with the changes in gene expression determined by microarray analysis. Together, our findings argue that aberrant expression of SOX2 in i-SOX2-DAOY cells limits their proliferation and induces the DAOY cells to exhibit markers of neural differentiation.

## Discussion

Improving our understanding of the fundamental mechanisms that govern cell proliferation and self-renewal is vital for the future development of therapeutic approaches for treating devastating cancers, such as glioblastoma and medulloblastoma. In this regard, studies to understand the basic biology of ESC have revealed that the transcription factor SOX2 is a central node in the vast gene regulatory networks necessary for maintaining the self-renewal and pluripotency of ESC. Moreover, studies conducted with ESC and NSC demonstrated that small changes in the expression of SOX2 dramatically alter their fate. In an important connection to these findings, SOX2 overexpression has been reported recently in many aggressive tumors, including tumors of the breast, lung, prostate and brain.

In this study, we demonstrate that elevation of SOX2 levels ∼2-fold in U87 and U118 glioblastoma cells leads to significant reductions in cellular proliferation by ∼65% (Figure 1) and ∼55% (Figure S1), respectively. Likewise, a 3-fold increase in SOX2 levels dramatically reduces DAOY medulloblastoma cell proliferation by ∼90% (Figure 2). Importantly, neurosphere studies conducted with freshly isolated CD133^−^ DAOY cells, as well as putative tumor-initiating, CD133^+^ DAOY cells, support the conclusion that SOX2 levels must be carefully regulated in order to maintain the proliferation of DAOY medulloblastoma cells (Figure 7).

In an effort to understand how elevating SOX2 retards the growth of DAOY cells, we probed for changes in cell-cycle distribution, gene expression and cell surface markers. In the case of the cell cycle, we observed significant changes 2 days after SOX2 levels were elevated (Figure 6D). Notably, there was a significant enrichment in the G1 compartment. Subsequent analysis demonstrated that this accumulation appears to reflect an increase in the proportion of quiescent cells in the population, rather than an increase in senescence as evidenced by a lack of β-galactosidase staining. Interestingly, RNA analysis indicated that elevation of SOX2 in i-SOX2-DAOY cells led to increases in the expression of gene markers associated with neural differentiation and reduced expression of markers associated with multipotent neural stem cells (Figure 8). Thus, our data suggest that ectopically elevating SOX2 in DAOY medulloblastoma cells causes them to undergo physiological changes that resemble a more differentiated phenotype.

In addition to significant changes in cell fate, cell cycle and gene expression, elevating SOX2 alters the proportion of the DAOY cell population that expresses the cell surface markers CD133 and SSEA1. Induction of exogenous SOX2 shifts the distribution of DAOY cells towards CD133 and/or SSEA1 expressing cells. Importantly, this finding suggests that CD133/SSEA1 low and high populations are not static, but that these populations are fluid and can interconvert. Moreover, our studies suggest that overexpression of SOX2 imparts a significant stress upon brain tumor cells. Interestingly, other studies have shown that brain tumor cells can dynamically alter the distribution of cells that express CD133 in response to changes in their culture environment, specifically changes in oxygen tension [36]. Together, our findings with SOX2 and those observed at low oxygen levels raise the possibility that CD133 plays an important role in the stress response of brain tumor cells in particular, and in cellular physiology in general.

Previous reports provide insight into regulatory mechanisms that may be essential for the careful regulation of SOX2 in brain tumors. The SOX2 gene promoter has been reported to be hypomethylated in clinical glioblastoma tumor samples, thereby enabling the expression of the SOX2 gene [12]. Thus, it is likely that regulatory mechanisms, which suppress the aberrant expression of SOX2 in other neural tissues, must be modified to support the long-term expression of SOX2 in brain tumor cells. Brain tumor cells expressing SOX2 also have regulatory mechanisms in place to restrain its aberrant expression. In this regard, miR-145 has been shown to block the translation of SOX2 transcripts in glioblastoma cells [37]. Additionally, SOX2 has been shown to be modified by a number of post-translational modifications, including phosphorylation [38], sumoylation [39], methylation [40], acetylation and ubiquitination [41], that may affect the localization, stability and function of SOX2. Understanding which mechanisms are used to control the expression and function of SOX2 will provide significant insight into the biological processes that are required by cancers employing SOX2 for their tumorigenicity.

Our findings with brain tumor cells raised an important question: do brain tumor cells respond differently to increases in SOX2 than other types of tumors? To address this question, we elevated SOX2 in prostate and breast cancer cells and observed that a 2- to 3-fold induction of SOX2 significantly reduced cell number *in vitro* (Figures 3 & 4). While our findings differ significantly from those reported by others [18], [19], it is likely that these differences are attributable to important differences in experimental design. In our studies, SOX2 was elevated from an inducible promoter in the total population of infected DU-145 prostate cancer, as well as MCF7 and MDA-231 breast cancer cells. Remarkably, these studies demonstrate that increased SOX2 levels are detrimental to the majority of the cell population soon after SOX2 is increased. Conversely, others isolated and then studied tumor cells that stably express elevated levels of SOX2 (∼3- to 4-fold) from a drug selected transgene. The differences observed in our studies and those of others appear to be due to the selection of a subpopulation of cells that not only tolerate elevated levels of SOX2, but exhibit enhanced proliferative capacity both *in vitro* and *in vivo* in response to elevated SOX2. This does not diminish the importance of SOX2 expression in prostate, breast or brain tumor cells. Rather, it strongly suggests that SOX2 levels in these tumors are unlikely to rise in isolation, but must be accompanied by other changes in gene expression that work in cooperation with SOX2 to enhance the growth of tumor cells. Importantly, we believe future studies should be directed toward the identification of changes in gene expression that work cooperatively with SOX2. Identifying genes required by SOX2 for growth promotion may offer new therapeutic targets for the treatment of SOX2-dependent cancers. Additionally, our findings illustrate the advantages offered by the use of inducible promoters when studying the roles of specific genes in cellular physiology.

In conclusion, the findings described in this study underscore the importance of carefully regulating the expression and function of the essential transcription factor SOX2 in brain tumor cells. Understanding how SOX2 levels are carefully controlled in tumor cells will provide novel insights into the fundamental biology of these cancers. Moreover, this understanding may help identify new targets for the development of improved therapeutic approaches to treat these devastating cancers.

## Supporting Information

Figure S1 **Ectopic elevation of SOX2 in U118 glioblastoma cells.** Photomicrographs of U118 cells infected with FUW-M2rtTA or FUW-M2rtTA and FUW-tetO-SOX2 lentiviruses, cultured in various concentrations of Dox. Photomicrographs were taken 48 hours after the addition of Dox to the culture medium. (B) MTT assay of U118 glioblastoma cells infected with FUW-M2rtTA or FUW-M2rtTA and FUW-tetO-SOX2 lentiviruses, cultured in various concentrations of Dox for 48 hours. Triplicates of each condition tested were averaged, and the error bars represent standard deviations. MTT values of cells cultured without Dox were set to one. This experiment was repeated two additional times, and similar results were obtained in each case. ‘*’ and ‘**’ indicate statistical significance (p<0.01 and p<0.001, respectively, student’s t-test). (C) Western blot analysis of SOX2 protein levels in nuclear extracts from U118 cells infected with FUW-M2rtTA and FUW-tetO-SOX2 lentiviruses, and cultured without or with Dox for 24 hours to induce SOX2 expression.
(TIF)

Figure S2 **Ectopic elevation of SOX2 in MDA-231 breast cancer cells.** (A) Photomicrographs of MDA-231 breast cancer cells infected with FUW-M2rtTA or FUW-M2rtTA and FUW-tetO-SOX2 lentiviruses, cultured in the presence of Dox, at the indicated concentration, for 48 hours. (B) MTT assay of MDA-231 breast cancer cells infected with FUW-M2rtTA or FUW-M2rtTA and FUW-tetO-SOX2 lentiviruses, cultured in various concentrations of Dox for 48 hours. Triplicates of each condition tested were averaged, and the error bars represent standard deviations. MTT values of cells cultured without Dox were set to one. This experiment was repeated two additional times, and similar results were obtained in each case. ‘*’ and ‘**’ indicate statistical significance (p<0.01 and p<0.001, respectively, student’s t-test). (C) Western blot analysis of SOX2 protein levels in nuclear extracts from MDA-231 cells infected with FUW-M2rtTA and FUW-tetO-SOX2 lentiviruses, and cultured without or with Dox for 24 hours to induce SOX2 expression.
(TIF)

Figure S3 **Elevated SOX2 levels impair neurosphere growth of DAOY cells.** Photomicrographs of DAOY medulloblastoma cells infected with FUW-M2rtTA or FUW-M2rtTA and FUW-tetO-SOX2 lentiviruses, cultured as neurospheres, in various concentrations of Dox. Photomicrographs were taken five days after cultures were set up.
(TIF)

Figure S4 **Long-term growth of i-SOX2-DAOY cells expressing exogenous SOX2.** i-SOX2-DAOY cells were grown in the absence and presence of Dox (1 µg/mL) for 4 weeks. Cells were subcultured weekly, at which time the Dox-treated and control populations were seeded at the same density. Photomicrographs of Dox-treated and control cells were taken at the end of the 4th week after being plated at the indicated cell densities (cells per T25 culture flask).
(TIF)

Figure S5 **Characterization of i-SOX2-U87 glioblasotma cells.** (A) Western blot analysis confirming Dox-inducible expression of flag-epitope tagged SOX2. i-SOX2-U87 cells were exposed to Dox for 24 hours and nuclear extracts were isolated. Photomicrographs of i-SOX2-U87 cells following (C) 72 and (D) 96 hours exposure ± Dox (1 µg/mL). (D) Determination of cell number was determined by MTT assay and direct cell counts with the aid of a Beckman Coulter Counter. In each case, three control samples and three Dox treated samples were analyzed. ‘*’ and ‘**’ indicate statistically significant difference (p<0.01 and p<0.001, respectively, student’s t-test) between untreated and Dox-treated samples. (E) Cell cycle analysis of i-SOX2-U87 cultured in the absence or presence of Dox (1 µg/mL) for 48 hours. Data for cells without Dox was collected as independent duplicates; whereas, samples with Dox were collected in triplicate. Error bars represent standard deviation. The student’s t-test was used to determine p-values. (F) Annexin V staining of i-SOX2-U87 cells cultured in the absence and presence of Dox for 48 hours.
(TIF)

Figure S6 **Long-term growth of i-SOX2-U87 cells expressing exogenous SOX2.** i-SOX2-U87 cells were grown in the absence and presence of Dox (1 µg/mL) for 4 weeks. Cells were subcultured weekly, at which time the Dox-treated and control populations were seeded at the same density. Photomicrographs of Dox-treated and control cells were taken at the end of the 4th week after being plated at the indicated cell densities (cells per T25 culture flask).
(TIF)

Table S1 **Primer sets used for RT-qPCR analysis.**
(XLS)

Table S2 **Microarray data from DAOY cells in the absence (−Dox) and presence (+Dox) of exogenous SOX2 expression.**
(XLS)

Methods S1 (DOC)
