# Supplementary figures and images for: Elevating SOX2 Levels Deleteriously Affects the Growth of Medulloblastoma and Glioblastoma Cells
Source: PLoS One. 2012 Aug 28;7(8):e44087. doi: 10.1371/journal.pone.0044087 (PMC3429438; doi:10.1371/journal.pone.0044087)

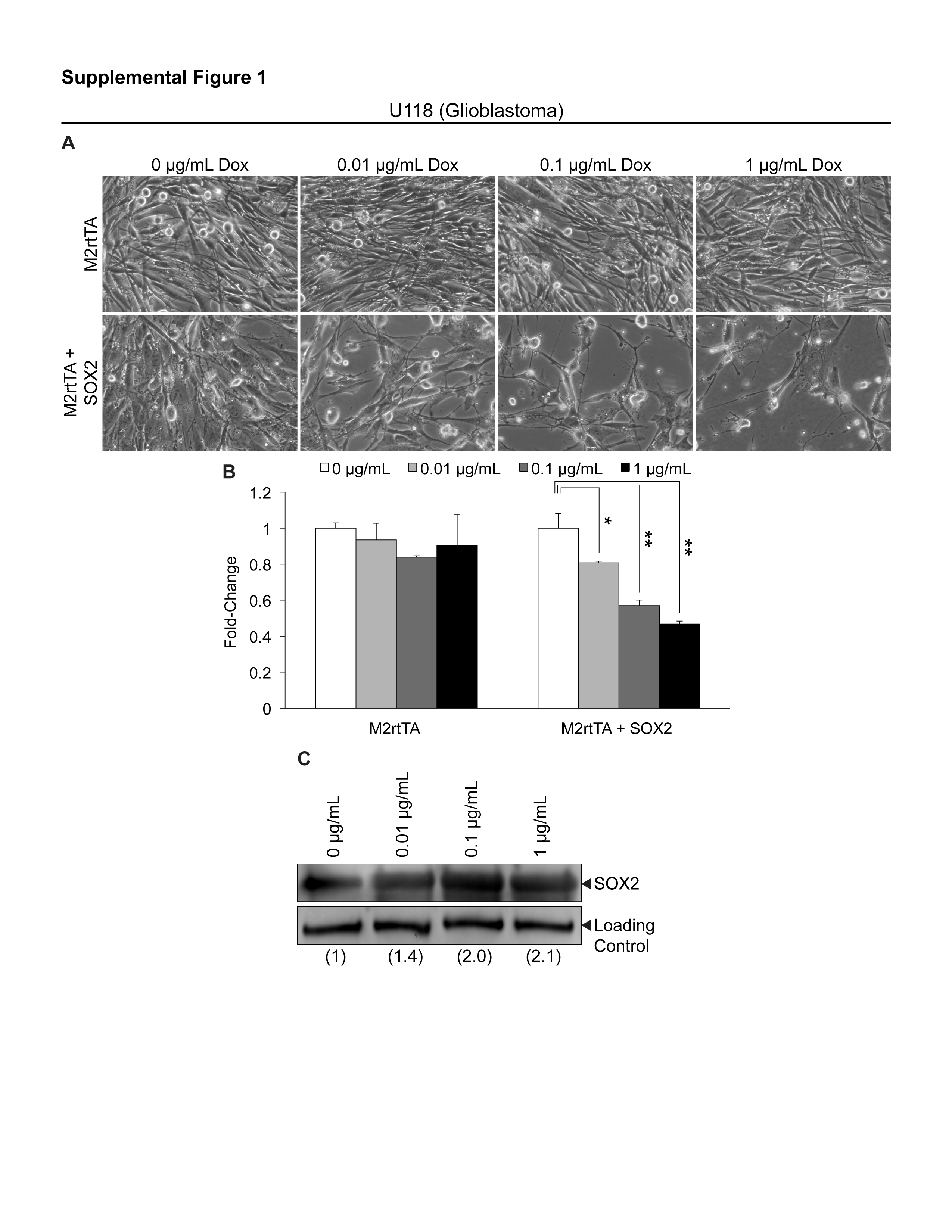

Supplement: Figure S1 — Ectopic elevation of SOX2 in U118 glioblastoma cells. Photomicrographs of U118 cells infected with FUW-M2rtTA or FUW-M2rtTA and FUW-tetO-SOX2 lentiviruses, cultured in various concentrations of Dox. Photomicrographs were taken 48 hours after the addition of Dox to the culture medium. (B) MTT assay of U118 glioblastoma cells infected with FUW-M2rtTA or FUW-M2rtTA and FUW-tetO-SOX2 lentiviruses, cultured in various concentrations of Dox for 48 hours. Triplicates of each condition tested were averaged, and the error bars represent standard deviations. MTT values of cells cultured without Dox were set to one. This experiment was repeated two additional times, and similar results were obtained in each case. ‘*’ and ‘**’ indicate statistical significance (p<0.01 and p<0.001, respectively, student’s t-test). (C) Western blot analysis of SOX2 protein levels in nuclear extracts from U118 cells infected with FUW-M2rtTA and FUW-tetO-SOX2 lentiviruses, and cultured without or with Dox for 24 hours to induce SOX2 expression. (TIF) [file pone.0044087.s001.tif]

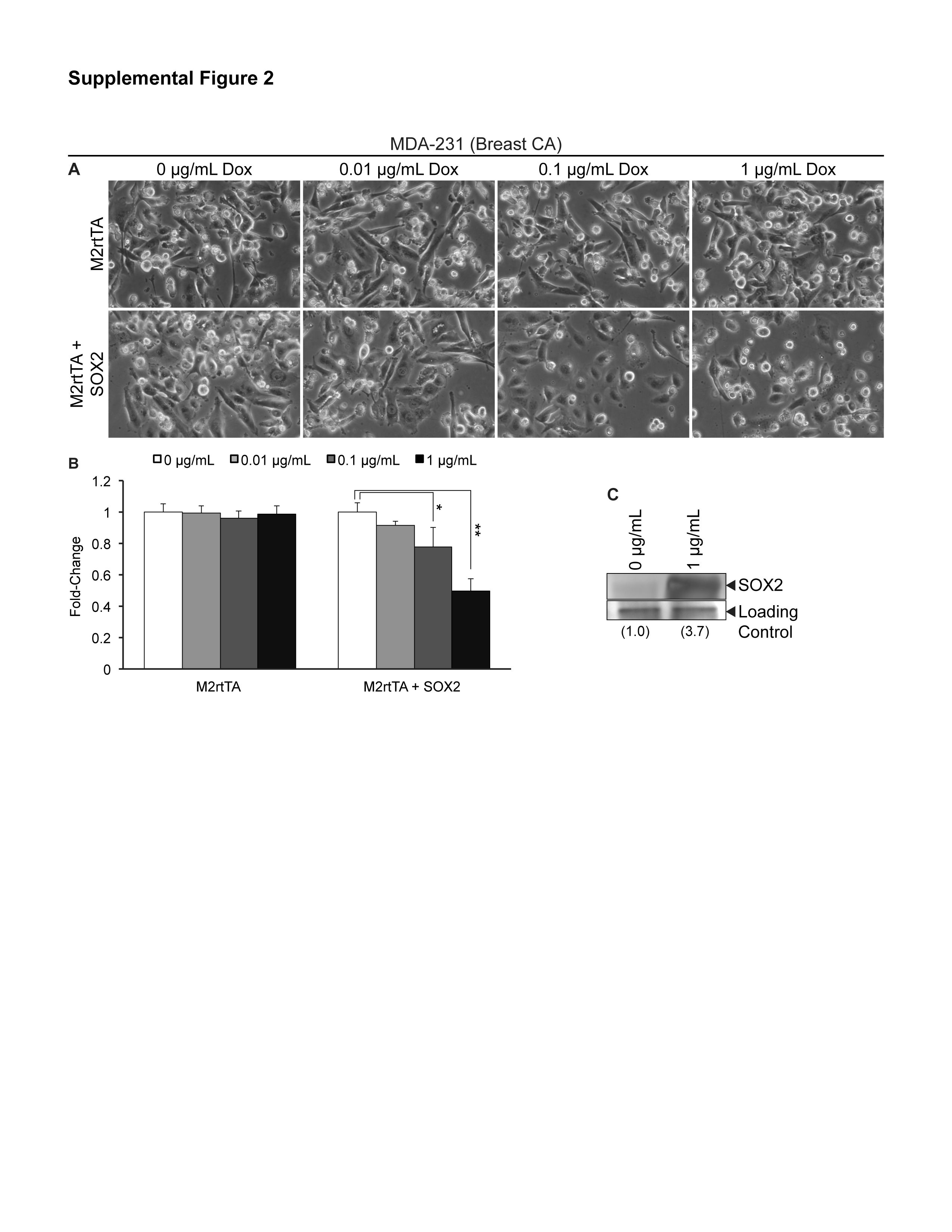

Supplement: Figure S2 — Ectopic elevation of SOX2 in MDA-231 breast cancer cells. (A) Photomicrographs of MDA-231 breast cancer cells infected with FUW-M2rtTA or FUW-M2rtTA and FUW-tetO-SOX2 lentiviruses, cultured in the presence of Dox, at the indicated concentration, for 48 hours. (B) MTT assay of MDA-231 breast cancer cells infected with FUW-M2rtTA or FUW-M2rtTA and FUW-tetO-SOX2 lentiviruses, cultured in various concentrations of Dox for 48 hours. Triplicates of each condition tested were averaged, and the error bars represent standard deviations. MTT values of cells cultured without Dox were set to one. This experiment was repeated two additional times, and similar results were obtained in each case. ‘*’ and ‘**’ indicate statistical significance (p<0.01 and p<0.001, respectively, student’s t-test). (C) Western blot analysis of SOX2 protein levels in nuclear extracts from MDA-231 cells infected with FUW-M2rtTA and FUW-tetO-SOX2 lentiviruses, and cultured without or with Dox for 24 hours to induce SOX2 expression. (TIF) [file pone.0044087.s002.tif]

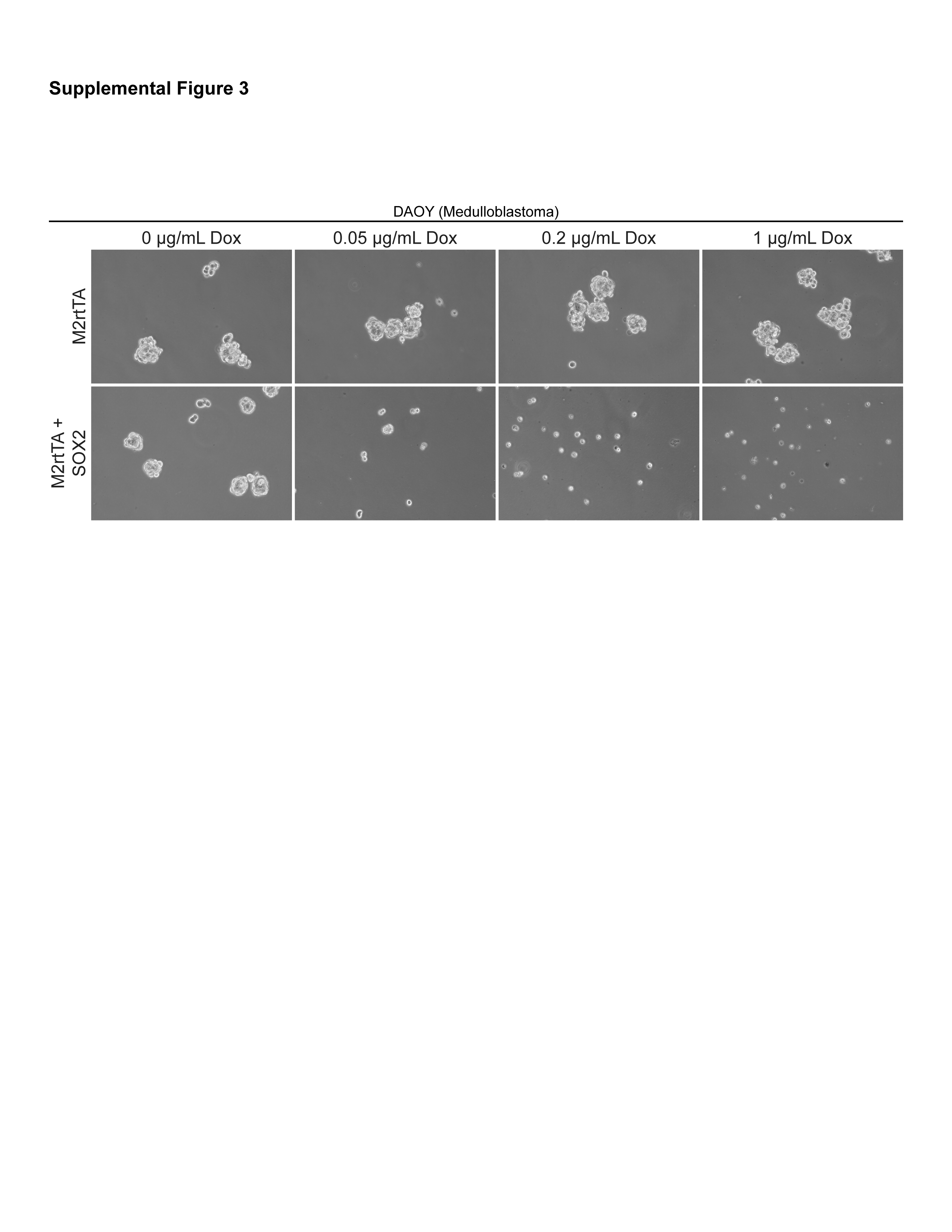

Supplement: Figure S3 — Elevated SOX2 levels impair neurosphere growth of DAOY cells. Photomicrographs of DAOY medulloblastoma cells infected with FUW-M2rtTA or FUW-M2rtTA and FUW-tetO-SOX2 lentiviruses, cultured as neurospheres, in various concentrations of Dox. Photomicrographs were taken five days after cultures were set up. (TIF) [file pone.0044087.s003.tif]

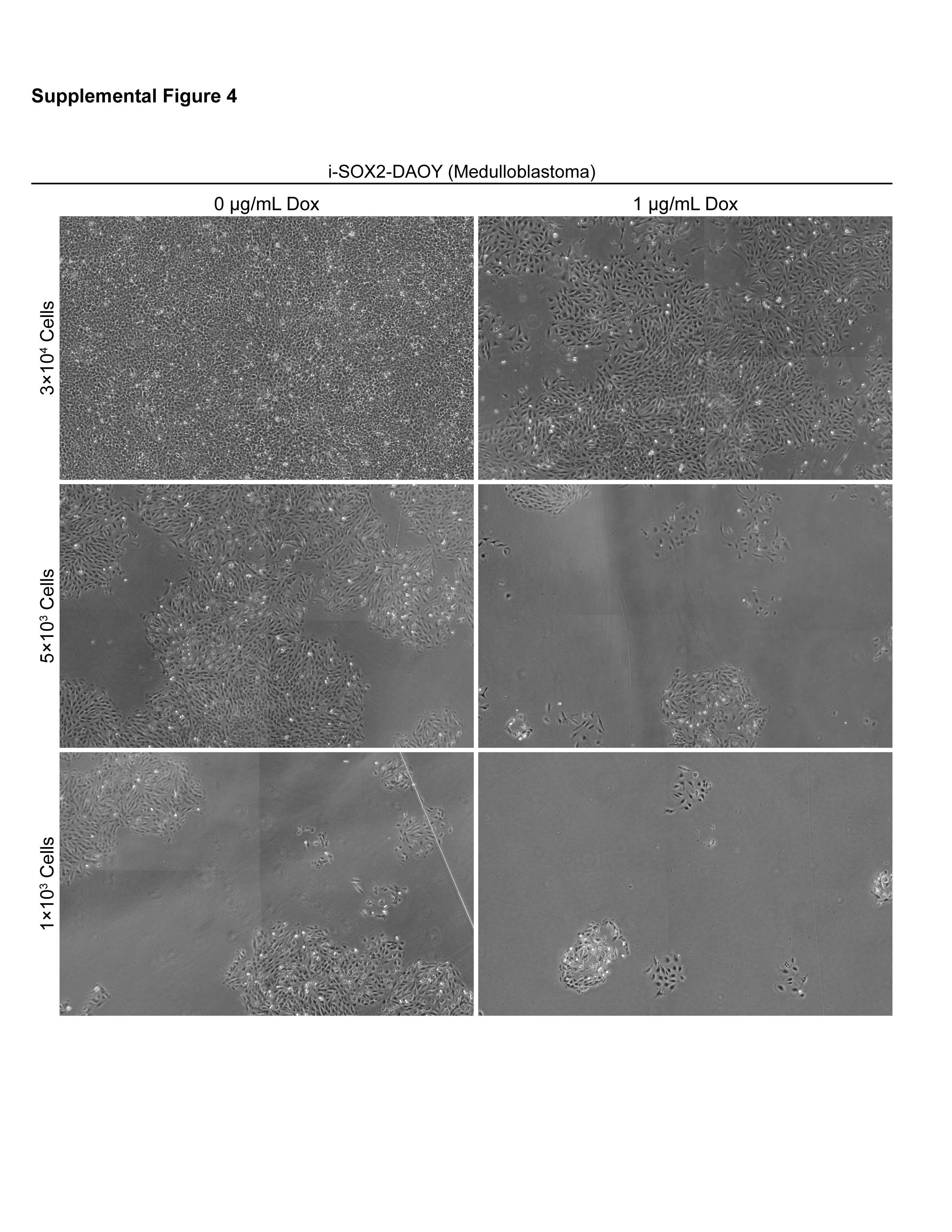

Supplement: Figure S4 — Long-term growth of i-SOX2-DAOY cells expressing exogenous SOX2. i-SOX2-DAOY cells were grown in the absence and presence of Dox (1 µg/mL) for 4 weeks. Cells were subcultured weekly, at which time the Dox-treated and control populations were seeded at the same density. Photomicrographs of Dox-treated and control cells were taken at the end of the 4th week after being plated at the indicated cell densities (cells per T25 culture flask). (TIF) [file pone.0044087.s004.tif]

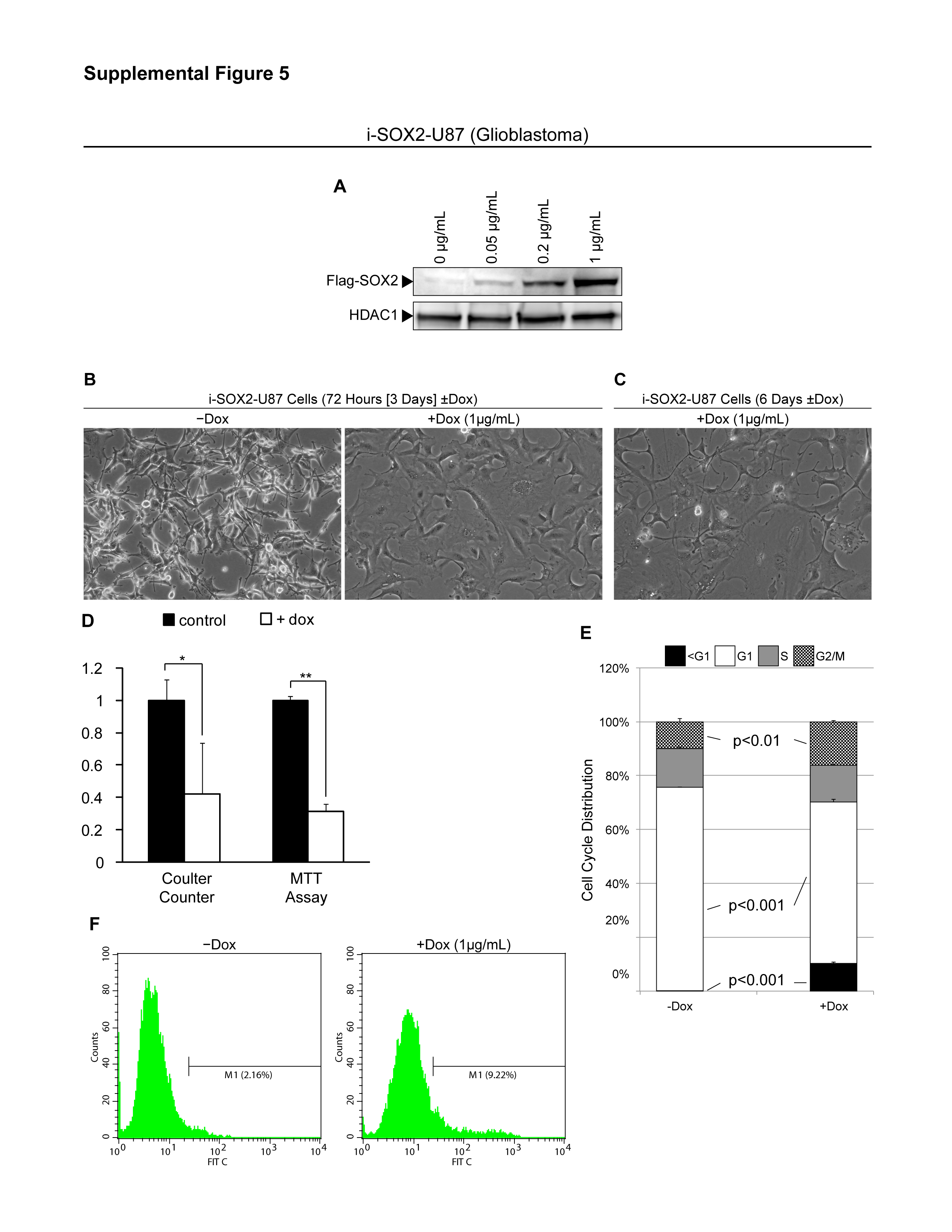

Supplement: Figure S5 — Characterization of i-SOX2-U87 glioblasotma cells. (A) Western blot analysis confirming Dox-inducible expression of flag-epitope tagged SOX2. i-SOX2-U87 cells were exposed to Dox for 24 hours and nuclear extracts were isolated. Photomicrographs of i-SOX2-U87 cells following (C) 72 and (D) 96 hours exposure ± Dox (1 µg/mL). (D) Determination of cell number was determined by MTT assay and direct cell counts with the aid of a Beckman Coulter Counter. In each case, three control samples and three Dox treated samples were analyzed. ‘*’ and ‘**’ indicate statistically significant difference (p<0.01 and p<0.001, respectively, student’s t-test) between untreated and Dox-treated samples. (E) Cell cycle analysis of i-SOX2-U87 cultured in the absence or presence of Dox (1 µg/mL) for 48 hours. Data for cells without Dox was collected as independent duplicates; whereas, samples with Dox were collected in triplicate. Error bars represent standard deviation. The student’s t-test was used to determine p-values. (F) Annexin V staining of i-SOX2-U87 cells cultured in the absence and presence of Dox for 48 hours. (TIF) [file pone.0044087.s005.tif]

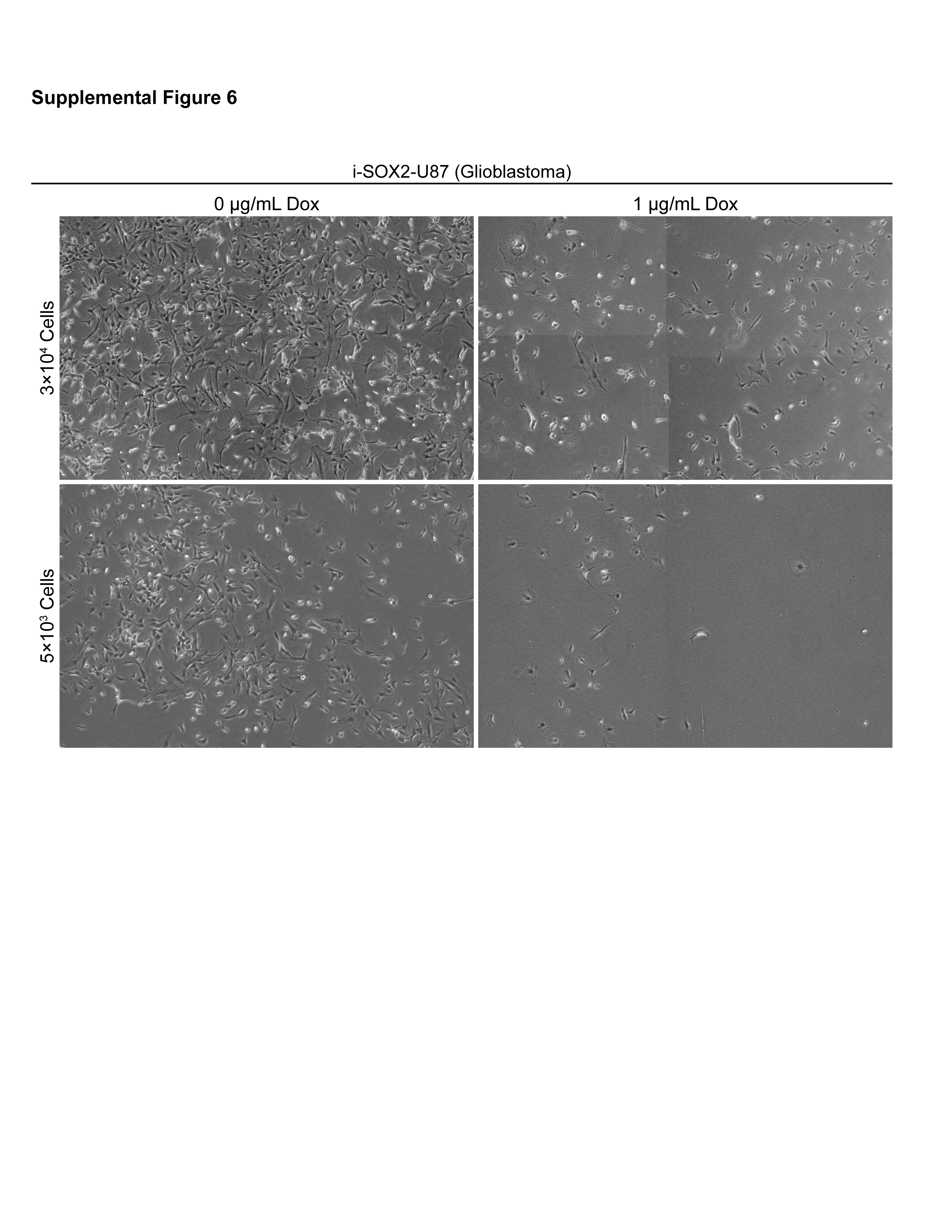

Supplement: Figure S6 — Long-term growth of i-SOX2-U87 cells expressing exogenous SOX2. i-SOX2-U87 cells were grown in the absence and presence of Dox (1 µg/mL) for 4 weeks. Cells were subcultured weekly, at which time the Dox-treated and control populations were seeded at the same density. Photomicrographs of Dox-treated and control cells were taken at the end of the 4th week after being plated at the indicated cell densities (cells per T25 culture flask). (TIF) [file pone.0044087.s006.tif]
